# P-1833. Acute Virulence Expression and Evolution of Artificial Airway Adapted *Pseudomonas aeruginosa*

**DOI:** 10.1093/ofid/ofae631.1996

**Published:** 2025-01-29

**Authors:** Glenn J Rapsinski, Vaughn Cooper, Anna Kozemchok

**Affiliations:** Children's Hospital of Pittsburgh of UPMC, Pittsburgh, Pennsylvania; University of Pittsburgh School of Medicine, Pittsburgh, Pennsylvania; Saint Vincent College, Greensburg, Pennsylvania

## Abstract

**Background:**

Mechanical ventilation is life-saving and may be lifelong in medically complex children with tracheostomy tubes (TT). Ventilator-associated infections (VAI), a common complication of mechanical ventilation, result in an estimated $1.25 billion per year of healthcare costs. *Pseudomonas aeruginosa* (Pa) frequently colonizes TT and endotracheal tubes and is a leading cause of VAI. Despite the importance of VAI, little is known about how Pa establishes colonization, limiting our ability to improve management. We hypothesize that Pa adaptively evolves to reduce its potential for acute virulence to facilitate colonization.

**Methods:**

Convenience samples of Pa isolates from patients requiring endotracheal tubes or TT were collected from the UPMC Children’s Hospital of Pittsburgh microbiology lab. Isolates were screened for virulence factors including proteases, rhamnolipids, twitching motility, lysis and sheen, amino acid auxotrophy, and mucoid colony morphology. Isolate infectivity was tested on a in vitro primary human cell epithelial model. DNA was extracted from longitudinal isolates for whole genome sequencing.

**Results:**

One hundred thirty-five isolates from 124 unique patients were screened for acute and chronic virulence phenotypes in a cross-sectional study. Low protease production, rhamnolipid production, and reduced twitching motility were more frequently present in respiratory isolates compared to skin and soft tissue infection (SSTI) isolates. Lysis and sheen were present in approximately one-third of respiratory isolates, similar to SSTI isolates. In longitudinal specimens from 23 patients, 95% of patients maintained their Pa genotype over time and representation from all three clades (PAO1, PA14, and PA7) of Pa were detected, with the PAO1 clade containing most isolates. Whole genome sequencing revealed mutations in global virulence regulators. Respiratory isolates had reduced infectious capabilities in an epithelial cell infection model.

**Conclusion:**

These data suggest that isolates from mechanically ventilated patients are diverse between patients, maintained within a given patient over time, and have lower virulence expression than Pa isolates from other sites.

**Disclosures:**

**All Authors**: No reported disclosures

